# Multisystem inflammatory syndrome in children

**DOI:** 10.3906/sag-2105-342

**Published:** 2021-12-17

**Authors:** Selda HANÇERLİ TÖRÜN, Dilek YILMAZ ÇİFTDOĞAN, Ateş KARA

**Affiliations:** 1 Department of Pediatrics, Pediatric Infectious Disease Unit, İstanbul University Faculty of Medicine, İstanbul Turkey; 2 Department of Pediatrics, Pediatric Infectious Disease Unit, Health Science University, İzmir Tepecik Research and Training Hospital, İzmir Turkey; 3 Department of Pediatrics, Pediatric Infectious Disease Unit, Hacettepe University Faculty of Medicine, Ankara Turkey

**Keywords:** Multisystem inflammatory syndrome, COVID-19, children

## Abstract

As the COVID-19 pandemic continues, *children* can be infected with the virus that causes *COVID*-19. Clinical symptoms of children with COVID from China, where the disease was first reported, generally were less severe than adults. However, at the end of April 2020 in Europe, it was observed that some children with SARS-CoV-2 infection developed fever, abdominal pain, shock, myocardial insufficiency and they needed to be taken care of in intensive care unit. This new disease has been called multisystem inflammatory syndrome in children (MIS-C). Although the pathogenesis of MIS-C is unclear, it progresses with signs of multiorgan involvement as a result of uncontrolled inflammation of the immune system and even causes death. Therefore, the diagnosis and treatment of patients with MIS-C should be managed quickly. In this review, the pathophysiology, clinical and laboratory findings, diagnostic methods, and treatment regimens of MIS-C were discussed.

## 1. Introduction

As the COVID-19 pandemic continues, it is becoming increasingly clear that severe clinical manifestations of SARS-CoV-2 infection remain rare in children, accounting for only 1.5% of all COVID-19 hospital admissions [1]. Clinical symptoms of children with COVID from China, where the disease was first reported, generally were less severe than adults [2]. However, on April 27, 2020, The Pediatric Intensive Care Society of the United Kingdom released an alert regarding an increased number of children presenting with fever, shock, abdominal pain, vomiting, and diarrhoea , with disease features that overlap with Kawasaki Disease (KD) and Toxic Shock Syndrome (TSS), many of whom had tested positive for COVID-19 [3]. This syndrome has been described in Europe as transient SARS-Cov-2 associated pediatric inflammatory multisystem syndrome (PIMS-TS), while Center for Disease Control and Prevention (CDC) reported that multiple systemic inflammatory syndrome is associated with COVID-19 (MIS-C), between March 1 and May 10, 2020 [4,5].

Children with MIS-C may have a fever and various symptoms, including abdominal pain, vomiting, diarrhea, neck pain, rash, bloodshot eyes, or feeling extra tired. Some features of MIS-C resemble KD, TSS, and secondary hemophagocytic lymphohistiocytosis/macrophage activation syndrome. Kawasaki disease is a syndrome of unknown cause that results in a fever and mainly affects children under 5 years of age. It is a form of vasculitis, where blood vessels become inflamed. Although classic Kawasaki’s disease disproportionately affects Asian children and young children, MIS-C that is associated with Covid-19 appears to occur among children of all ethnic backgrounds and is detected in older children [6]. The relationship of MIS-C to SARS-CoV-2 infection suggests that the pathogenesis involves post-infectious immune dysregulation. However, the possibility that MIS-C is more consistent with a subacute infection than a post-infectious syndrome was not fully considered.

## 2.Epidemiology

In April 2020, cases resembling incomplete Kawasaki disease or toxic shock syndrome thought to be associated with COVID-19 were reported first time from UK [3]. Then, an increase was observed in the reporting of similar cases from various countries around the world, especially in New York (USA). Based on epidemiological data from the UK, New York, and Italy, it was also noted that there was a delay of several weeks between when COVID-19 cases were most common in the community and when the number of MIS-C cases was the highest. Epidemiological data clearly indicate that SARS-CoV-2 is the trigger for MIS-C, which typically occurs about 1 month after infection. 

As of October 1, 2020, the number of patients meeting the case definition for MIS-C in the United States surpassed 1000. In 2021, this number surpassed 2000 as of 2021 February 1 and 3000 as of April 1 [7]. In USA, a descriptive study showed that the peak in the number of MIS-C cases followed the peak in the number of cases of laboratory-confirmed SARS-CoV-2 infection by 31 days. From March 1 through May 10, 2020, the incidence of laboratory-confirmed SARS-CoV-2 infection was 322 per 100,000 persons younger than 21 years of age, and the incidence of MIS-C was 2 per 100,000 persons younger than 21 years of age [8].

There has been a striking paucity of MIS-C cases from East Asia. Only two cases of MIS-C have been reported to date in South Korea During May–November 2020; a total of 2287 COVID-19 cases in persons 0–19 years of age was reported, and no cases have been reported in Japan or China as opposed to the higher number of cases from the Americas, Europe, Africa, South Asia, and the Middle East [9,10].

## 3.Pathophysiology

The reasons for the milder course of SARS-CoV-2 infection in children compared to adults have been investigated, but the reasons have not been clearly determined. Several theories have been discussed involving differences in the immune system, such as thymic function difference, cross-reactive immunity against other coronaviruses, as well as differences in the expression of the angiotensin converting enzyme 2 (ACE2) receptor that the virus uses to enter the cell [11]. The immune system undergoes substantial changes from birth to adulthood [12]. In a study, it has been reported that patients with MIS-C have ineffective and reduced neutralizing antibody activity against SARS-Cov-2 compared to adults who have severe COVID-19 and recovered [13].

Generally, viruses are not detected in the respiratory tract of patients with MIS-C, but other parts of the body, such as the gastrointestinal tract, have not been studied sufficiently [14]. A recent study revealed the presence of SARS-CoV-2 in lungs, heart, kidneys by at least one method (RT-PCR, IHC, or EM), and in endothelial cells of heart and brain in two patients with MIS-C (IHC). And 2 in the brain tissue of a child with MIS-C with acute encephalopathy, and in the intestinal tissue of a child with acute colitis [15]. The presence of SARS-CoV-2 in several organs, associated with cellular ultra-structural changes, reinforces the hypothesis that a direct effect of SARS-CoV-2 on tissues is involved in the pathogenesis of MIS-C. According to Schwarz’s hypothesis, there may be persistent infection [16]. This hypothesis said that in MIS-C, immune responses that have not fully controlled an ongoing infection might allow persistent activation of the intrinsic immune system. This persistent innate immune inflammatory response probably occurs because of the ability of SARS-CoV-2 to block type 1 and type 3 interferon response signalling to the adaptive immune system without disrupting cytokine production. If MIS-C is confirmed to be caused by ongoing infection, this finding might also have important implications for antiviral treatment of adults with prolonged symptoms after SARS-CoV-2 infection.

In another study, the inflammatory differences observed in children with KD and MIS-C such as interleukin-17A mediated hyperinflammation response. Additionally, differences were found in T cells subsets and cytokine mediators. It was found that diffuse endothelial involvement was higher in MIS-C than KD. In this study, autoantibody formation hypothesis was proposed as the cause of this immune response, and it was thought that these autoantibodies contributed to damage in MIS-C [17]. Some studies investigate the therapeutic potential of peptide mimetics of SARS-CoV-2 spike SAg-like region in COVID-19-induced hyperinflammatory syndrome similar to toxic shock syndrome (TSS) [18].

Angiotensin-converting enzyme 2 receptor is expressed in the heart, kidneys and testicles, and lungs. It is commonly found in human alveolar epithelial cells, small intestine epithelial cells, arterial smooth muscle, and vascular endothelial cells [19]. Increasing evidence suggests that ACE2 enzyme activity has a protective role in cardiovascular disease; ACE2 loss can be harmful because it can lead to worsening of heart function and progression of cardiovascular disease [20]. Therefore, ACE2 receptor loss due to SARS-CoV-2 infection may lead to myocardial damage [21,22].

## 4.Clinical presentation

Clinical presentation of MIS-C is dominated by significant inflammation. Most affected children are previously healthy and have no history of comorbid diseases (Table 1). However, it is noteworthy that the rate of obese patients has increased in some studies [23,24]. 

**Table 1:** Demographic and clinical characteristics of MIS-C patients announced by CDC (38).

Clinical characteristics	Total cases (%)	Clinical characteristics	Total cases (%)
Total cases	570 (100)	CardiovasculerShockTroponin elevationElevated BNP/NT-Pro BNP Congestive heart failureCardiac dysfunctionMiyocarditis Coronary artery dilatation HypotensionPericardial effusion Mitral regurgitationDermatologic/ mucocutaneousRashMucocutaneous lesionsConjunctival injectionHematologic Elevated D–dimerThrombocytopeniaLymphopeniaRespiratoryCoughShortness of breathChest pain or /tightnessPneumonia†ARDSPleural effusion§Neurologic HeadacheRenalAcute kidney injuryOtherPeriorbital edemaCervical lymphadenopathy >1.5 cm diameterGastrointestinal symptomAbdominal painVomitingDiarrhea	493 (86.5)202 (35.4)176 (30.9)246 (43.2)40 (7)207 (40.6)130(22.8)95 (18.6)282 (49.5)122 (23.9)130 (25.5)404 (70.9)315 (55.3)201 (35.3)276 (48.4)421 (73.9)344 (60.4)176 (30.9)202 (35.4)359 (63)163 (28.6)149 (26.1)66 (11.6)110 (19.3)34 (6)86 (15.8)218 (38.2)186 (32.6)105 (18.4)105 (18.4)27 (4.7)76 (13.3)518 (90.9)353 (61.9)352 (61.8)303 (53.2)
SexFemaleMale	316 (55.4)254 (44.6)		
Age (year), median (IQR)	8 (4–12)		
Death	10 (1.8)		
Hospitalization (day) IQR1 2-7 8-14 ≥ 15 Unknown	6 (4-9)16 (3.2)304 (60.2)149 (29.5)36 (7.1)65 (-)		
PICU	364 (63.9)		
Hospitalization of PICU(day) (IQR)	5 (3–7)		
ObeziteChronic lung disease	146 (25.6%)48 (8.4%)		
No. of organ systems involved2-34-5≥6	80 (14.0%)351 (61.6%)139 (24.4%)		


Abbreviations: ARDS = acute respiratory distress syndrome; BNP = brain natriuretic peptide; PICU =pediatric  intensive care unit; IQR = interquartile range; NT-proBNP = N-terminal pro b-type natriuretic peptide; PCR = polymerase chain reaction.Fever is the most important finding found in MIS-C. Presence of resistant and prolonged fever is different from many diseases [25]. In addition to the presence of fever, gastrointestinal symptoms, including vomiting, abdominal pain and / or diarrhea, mucocutaneous symptoms, including conjunctivitis and rash reminiscent of KD, and symptoms such as headache, irritability, and encephalopathy may also include neurological symptoms [26,27]. These findings are nonspecific and may occur with noninfectious etiologies such as oncological or inflammatory conditions, as well as other infectious diseases. More rarely, cases who underwent laparotomy for reasons such as mesenteric lymphadenitis and peritonitis have been reported [28]. In some patients, respiratory distress due to hypotension requires inotropic support, and, in others, due to cardiac dysfunction, it requires noninvasive or invasive mechanical ventilation, and even requires admission to the pediatric intensive care unit. In a patient with suspected multiple systemic inflammatory disease, if there is a persistent fever and a history of exposure to the SARS-CoV2 virus, the presence of at least two of the listed findings is recommended to perform further investigations to show the diagnosis of MIS-C. These findings are as follows: rash (may be maculopapular and / or polymorphic and / or petechial rash; vesicular rash is not expected) gastrointestinal complaints (diarrhea, abdominal pain, vomiting) hand-standing edema, oral mucosa changes (red and / or cracked lips, strawberry tongue or oropharyngeal mucosa erythema), conjunctivitis (non-bilateral exudative), lymphadenopathy and neurological symptom (altered mental perception, encephalopathy, focal neurological findings, meningismus or papilledema) [27]. In the study of Whittaker et al. consisting of 58 cases, all of the patients presented with fever lasting 3–19 days, variable combinations of sore throat (n=6, 10%), headache (n=15, 26%), abdominal pain (n=31, 53%) and erythematous rash (n=30, 52%). More rarely, cases with conjunctivalerythema (n=26, 45%), lymphadenopathy, mucous membrane changes, red chapped lips, swelling in the hands and feet have also been reported. In another study, fever (> 38.5 °C) and malaise were found in all patients, while gastrointestinal symptoms were found in 80% of the patients. Among the symptoms suggestive of KD, skin rash, ileitis, cervical lymphadenopathy, and meningismus were observed to be frequent, and all of them met the criteria for complete KD [24]. In a study conducted in the United States of America, fever was found in all 186 MIS-C cases, most of the cases had gastrointestinal system (92%) complaints, and cardiovascular involvement (80%) was found to be common in the cases [25]. It was observed that 10 patients were admitted to the hospital with a fever lasting an average of six days, and half of the patients had classical KD findings, while the other half had incomplete KD findings. In addition, clinical signs of hypoperfusion and hypotension meeting KDSS criteria were detected in 5 cases. Cardiac manifestations are common, including ventricular dysfunction, coronary artery dilation and aneurysms, arrhythmia, and conduction abnormalities [29].

Studies show that the rate of intensive care hospitalization varies between 30%–80% [27,28,30]. Similarly, the need for inotropes was found to be 30%–50% in shock rates. Mortality rates vary; while death was reported in four (2%) cases in Feldstein et al.’s study, death was reported in five cases (11%) in the study of Mamishi et al. [25,31]. In addition, there are studies in which no deaths were reported [27,30]. The clinical features of severe acute COVID-19, and MIS-C may overlap. But different clinical findings and organ system involvement may be helpful at this point [32,33,34].

Most pediatric cases of severe acute COVID-19 occur in children with underlying health problems; however, most of the children with MIS-C were previously healthy. Respiratory symptoms are rare in patients with MIS-C but are more common in patients with severe acute COVID-19. Also, gastrointestinal symptoms, mucocutaneous findings, myocardial dysfunction, and shock are more common in MIS-C than in severe acute COVID-19.

## 5.Laboratory findings

Laboratory findings are variable. In a metaanalysis of 66 studies in children that included 9335 children with documented SARS-CoV-2 , the laboratory abnormalities were (mean proportion) as follows[35]:

●Elevated C-reactive protein (CRP) – 54%

●Elevated serum ferritin – 47%

●Elevated lactate dehydrogenase – 37%

●Elevated D-dimers – 35%

●Elevated procalcitonin – 21%

●Elevated erythrocyte sedimentation rate – 19%

●Elevated leukocytes – 20%

●Lymphocytopenia – 19%

●Lymphocytosis – 8%

●Elevated serum aminotransferases – 30%

●Elevated creatine kinase myocardial band – 25%

An exaggerated response may indicate MIS-C. Serum C reactive protein (CRP), erythrocyte sedimentation rate (ESR), fibrinogen, D-dimer, ferritin, lactatedehydegenase (LDH), interleukin 6 (IL-6) are increased; lymphopenia, thrombocytopenia, hypoalbuminemia and hyponatremia can be detected. In Whittaker et al.’s study, the mean CRP value (229 mg/L) was found to be very high [27]. In another study, the mean ESR was 72 mm/h, and the mean ferritin value was 1176 ng/mL. Eight of the cases (84.5%) had lymphopenia and hyponatremia, while seven (87%) had mild transaminase elevation (aspartate aminotransferase 87 U / L [SD 70]; alanine aminotransferase 119 U / L [ SD 217]), and triglyceride elevation. (239 mg / dL [SD 108]) was detected. In addition, increased fibrinogen (621 mg / dL [182]) was observed in nine cases (90%) and increased D-dimer (3798 ng / mL [SD 1318]) in eight cases [30].

 Electrocardiogram (ECG), cardiac enzymes (troponin, B-type natriuretic peptide [BNP] / N-terminal-pro-B-type natriuretic peptide [NT-proBNP]) and echocardiography are recommended for cardiac evaluation. In a study evaluating cardiac enzyme elevation, it was observed that trop824onin elevation was observed in 68% (34/50) of the cases, while NT-proBNP increased in 83% [30]. 

MIS-C associated with COVID-19 is characterized predominantly by cardiovascular abnormalities, although solid visceral organ, gall bladder, and bowel abnormalities, as well as ascites, are also seen, reflecting a multisystemic inflammatory process. Blumfield et al.’s study revealed that cardiomegaly (63%), congestive heart failure or cardiogenic pulmonary edema (56%), atelectasis (56%), pleural effusions (44%), acute respiratory distress syndrome (13%), and pneumonia (6%) were shown on chest radiography and abdominal imaging findings (ultrasound, CT, and radiography), together with small-volumea scites (38%), hepatomegaly (38%), echogenic kidneys (31%), bowel wall thickening (19%), gall bladder wall thickening (19%), mesenteric lymphadenopathy (13%), splenomegaly (16%), and bladder wall thickening (6%) [28].

## 6.Diagnosis

The MIS-C diagnostic criteria announced by the World Health Organization and CDC are summarized in Table 2 [4,36]. The definitions require patients to be less than 21 years old (CDC criteria) or less than 19 years old (WHO criteria) and to have evidence of recent or current SARS-CoV-2 infection or exposure (CDC only), the presence of documented fever, elevated markers of inflammation, at least two signs of multisystem involvement. Evaluation of a diagnosis for MIS-C must include research for other possible causes. This disease is temporarily associated with SARS-CoV-2 infections and has been reported as clusters of cases 2–6 weeks after the highest incidence of COVID-19 cases, typically in geographic areas with COVID-19 disease burden. In MIS-C, the timing of onset of symptoms is variable. The usual duration between acute SARS-CoV2 infection and onset of MIS-C symptoms is 2 to 6 weeks. However, in literature, rare cases of MIS-C occurring >6 weeks after the acute infection have been reported [37]

**Table 2:** Diagnostic Criteria for MIS-C

Centers for Disease Control and Prevention (CDC) (4)	World Health Organization (WHO) (36)
· An individual aged <21 years having fever*, laboratory evidence of inflammation**, and evidence of clinically severe illness requiring hospitalization, with multisystem (>2) organ involvement (cardiac, renal, respiratory, hematologic, gastrointestinal, dermatologic, or neurological); AND· No alternative plausible diagnoses; AND· Positive for current or recent SARS-CoV-2 infection by RT-PCR, serology, or antigen test; or exposure to a suspected or confirmed COVID-19 case within the 4 weeks prior to the onset of symptoms.*Fever >38.0°C for ≥24 h, or report of subjective fever lasting ≥24 h**Including, but not limited to, one or more of the following: an elevated C-reactive protein (CRP), erythrocyte sedimentation rate (ESR), fibrinogen, procalcitonin, d-dimer, ferritin, lactic acid dehydrogenase (LDH), or interleukin 6 (IL-6), elevated neutrophils, reduced lymphocytes and low albumin.	- Children and adolescents 0–19 years of age with fever > 3 daysAND two of the following:1. Rash or bilateral non-purulent conjunctivitis or muco-cutaneous inflammation signs (oral, hands or feet).2. Hypotension or shock.3. Features of myocardial dysfunction, pericarditis, valvulitis, or coronary abnormalities (including ECHO findings or elevated Troponin/NT-proBNP).4. Evidence of coagulopathy (by PT, PTT, elevated d-Dimers).5. Acute gastrointestinal problems (diarrhoea, vomiting, or abdominal pain).ANDElevated markers of inflammation such as ESR, C-reactive protein, or procalcitonin. ANDNo other obvious microbial cause of inflammation, including bacterial sepsis, staphylococcal or streptococcal shock syndromes.ANDEvidence of COVID-19 (RT-PCR, antigen test or serology positive), or likely contact with patients with COVID-19.

All patients with suspected MIS-C should demonstrate exposure to the SARS-CoV-2 virus. RT-PCR test should be performed to detect the SARS-CoV-2 virus at the time of application. If initially RT-PCR is detected negative, at least 24 h after the second test. In studies, it was observed that most of the cases (80%–90%) were positive for SARS-CoV-2 antibodies, RT-PCR positivity, and antigenicity was found to be positive in very few (20%–40%) cases [38,39].

Acute phase response patients with suspected MIS-C should demonstrate exposure to the SARS-CoV-2 virus. RT-PCR test should be performed to detect the SARS-CoV-2 virus at the time of application. Initially RT-PCR is detected negative, at least 24 h after a second test application. In studies, it was observed that most of the cases (80%–90%) were positive for SARS-CoV-2 antibodies, RT-PCR positivity and antigenicity were found to be positive in very few (20%–40%) of the cases [32].

## 7.Differrential diagnosis

MIS-C may show similar features with childhood diseases such as sepsis, KH, KDSS, macrophage activation syndrome (MAS), and TSS [33]. Sepsis is an important condition in children presenting with fever, shock, and high inflammatory markers. Blood cultures should be sent from all children with moderate to severe suspicion of MIS-C, and empirical antibiotics should be administered while the culture results are awaited. Some clinical features may help distinguish MIS-C from bacterial sepsis. For example, cardiac involvement, especially coronary artery involvement, is rare in bacterial sepsis. Also, microbiological tests (SARS-CoV-2 antigen / antibody / RT-PCR tests, bacterial cultures) are required to differentiate the disease.

Kawasaki disease is a vasculitis that usually occurs in young children and involves medium-sized vessels such as coronary arteries. It is the most common cause of childhood acquired heart disease in developed countries; features include rash, cervical lymph node enlargement, ocular and oral mucosal changes, hand-foot changes, as well as fever lasting at least five days. Apart from this, liver, lungs, gastrointestinal system, central nervous system and joints may be involved. Some clinical and laboratory findings may be useful in differentiating MIS-C and KD. Increased acute phase responses are seen in Kawasaki disease similar to MIS-C, while thrombocytosis is more common. Prominent abdominal pain and lymphopenia are more likely revealed in MIS-C disease [30]. In addition, patients with MIS-C are much more likely to exhibit cardiac dysfunction and especially hypotension compared to patients with KD. One of the cardiac markers, NT-ProBNPKH, is a potential marker and can be detected at levels as high as 7000 pg / mL in MIS-C. Values exceeding 10000 pg / mL are frequently reported (31). It is thought that Kawasaki disease is triggered by a viral infection, which is likely to occur in winter and spring months and causes an inflammatory response in genetically susceptible children (32). Kawasaki disease shock syndrome less than 5% of children with KD present with bacterial disease-like shock and hypotension and have higher neutrophil and band counts, lower platelet counts, lower hemoglobin levels, and higher CRP levels compared to KD (Table 3).

**Table 3:** Clinical characteristics of Kawasaki Disease and MIS-C

	Kawasaki Disease	MIS-C
Age	1–4 yearsold	Mean 8 years old (2-17 y)
Presenting symptoms	Fever for 5 days plus 4/5: conjunctivitis,rash,adenopathy, strawberry tounge,hand/food swelling	Persistent fever >24 h, GI symptoms, rash, conjunctivitis
Rash	Yes; polymorphus	Yes, less common to have mucosal involment
Gastrointestinal symptoms	Less common	Very common (abdominaş pain, vomiting, diarrhea
Labs	Leukocytosis, Trombocytosis, elevated CRP,ESR,	Lympocytopenia, Trombocytopenia,Elevated CRP, ESR, elevated cardiac markers
Echocardiyogram	Coronory artery abnormalities	Decreased left ventriculer function, coronoary artery abnommalities
Treatment	IVIG, aspirin	Generally supportive; anticoagulant, steroids, IL-1/IL-6 antagonists

Toxic shock syndrome is caused by uncontrolled activation of the immune system due to superantigens that stimulate T cells nonselectively. It is caused by bacterial species such as *Staphylococcus aureus *and *Streptococcus pyogenes*. The clinical picture of toxic shock syndrome was hypotension, diffuse erythrodermic rash, diffuse mucosal involvement, and multiple systemic dysfunction (renal, hepatic, hematological, respiratory, muscular, and neurological). Toxic shock syndrome may be similar to KDSS, but patients with TSS tend to be older (9 ± 4.6 years and 3 ± 3.4 years, respectively). In addition, TSS is more likely to have normal hemoglobin levels and low platelet counts [31].

Hemophagocytic lymphohistiocytosis (HLH) is a disease that develops secondary to anomalies in genes regulating the degranulation of natural killer cells and cytotoxic CD8 + lymphocytes, leading to a “cytokine storm” by eliminating antigenic stimuli that lead to cellular activation. High levels of proinflammatory cytokines, then, activate other cells of the immune system (macrophages), leading to organ damage and characteristic hemophagocytosis of affected organs. Secondary HLH (MAS) is triggered by an autoimmune or autoinflammatory condition; drugs, malignancy or infections, and especially viral infections are well-known triggers. In macrophage activation syndrome, there is typically high levels of evidence of systemic inflammation. Organ dysfunction such as coagulopathy, liver failure, central nervous system dysfunction, and cardiac dysfunction can be seen with CRP, triglycerides, and D-dimer. Especially in patients with MAS, leukocyte count, thrombocyte count, and ESR tend to be suppressed [38].

Other viral pathogens that should be considered in differential diagnosis with multiple systemic inflammatory syndrome include Epstein-Barr virus, cytomegalovirus, adenovirus, and enteroviruses; in these infections, they may present with multiple systemic involvement and / or myocarditis. These viruses rarely cause severe multisystemic involvement, especially in immunocompromised children. Serological tests and RT-PCR can be used for this purpose.

## 8.Treatment

The aim of treatment in MIS-C is to reduce systemic inflammation and restore organ function. By definition, MIS-C is a multisystem disease, and caring for affected children requires the coordination of many different specialties. These are as follows:

-Pediatric infectious diease specialist,

-Pediatric rheumatology specialist,

-Pediatric cardiolojist,

-Pediatric intensive care specialist,

-Pediatric hematology specilaist.

 Treatment depends on the clinical condition of the patient. Some interventions, such as empirical antibiotics, intravenous immune globulin (IVIG), and prophylactic antithrombotic therapy are suitable for most patients with moderate to severe symptoms [38]. The American Society of Rheumatology recommends routine IVIG treatment in cases with a diagnosis of moderate and severe MIS-C and also recommends IVIG for all patients with signs of Kawasaki disease. IVIG is also recommended in situations involving any of the following:

-Shock,

-Cardiac involvement, including any of the following;

-Supression of left ventricular function on echocardiolography,

-Coronary artery abnormalities (dilatation or aneurysm),

-Arrhytmia,

-High BNP/NT-Pro BNP and/or troponin levels.

-Other serious symptoms requiring pediatric intensive care unit.

**Figure 1:**
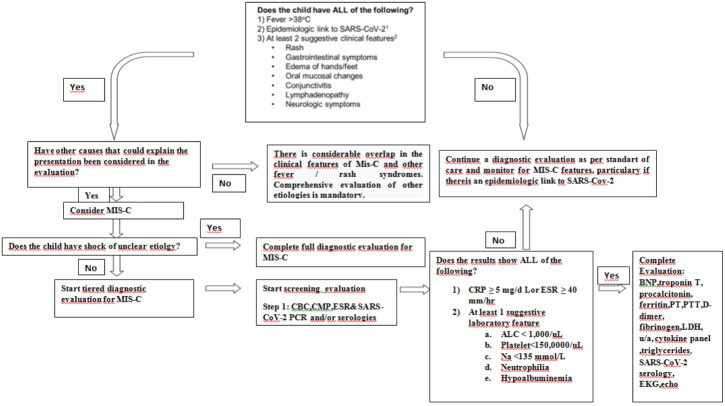
Clinical aproach to possiple MIS-C. Adapted from reference 32: Henderson LA, Canna SW, Friedman KG, Gorelik M, Lapidus SK, Bassiri H, et al. American College of Rheumatology Clinical Guidance for Multisystem Inflammatory Syndrome in Children Associated With SARS-CoV-2 and Hyperinflammation in Pediatric COVID-19: Version 2. Arthritis & rheumatology (Hoboken, NJ). 2021;73(4):e13-e29

 Patients who are being investigated for multiple systemic inflammatory syndrome should first undergo diagnostic evaluation if there is no life-threatening condition (Figure 1). These cases should be investigated before immunomodulatory therapy in terms of MIS-C as well as other possible infection and noninfectious related conditions. However, if there are life-threatening symptoms in the patients, it may be necessary to start immunomodulatory treatment without waiting for the completion of the full diagnosis evaluation [32].

**Figure 2:**
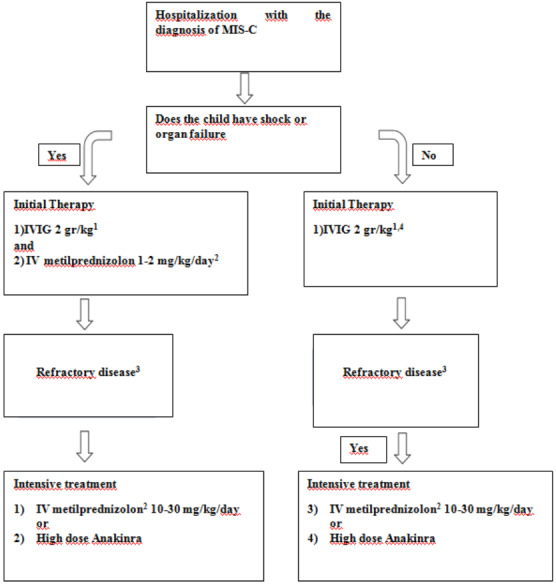
Clinical follow-up of MIS-C. Adapted from reference 32: Henderson LA, Canna SW, Friedman KG, Gorelik M, Lapidus SK, Bassiri H, et al. American College of Rheumatology Clinical Guidance for Multisystem Inflammatory Syndrome in Children Associated With SARS- CoV-2 and Hyperinflammation in Pediatric COVID-19: Version 2. Arthritis & rheumatology (Hoboken, NJ). 2021;73(4):e13-e29

Step-by-stepprogression of immunomodulatory therapies should be used to treat MIS-C (Figure 2). Intravenous immunoglobulin (IVIG) is considered first-line immunomodulatory therapy. Patients with features similar to Kawasaki disease without shock and cardiac involvement can initially be followed up conservatively. However, IVIG is typically recommended if the patient’s clinical condition worsens or in the presence of persistent fever or elevated inflammatory markers, including elevated ferritin levels [38].

 Glucocorticoids should be used as adjunctive therapy in patients with severe disease or as intensification therapy in patients with refractory disease. Intravenous immunoglobulin should be given to patients who are hospitalized and / or fully meet the KD criteria. High-dose IVIG (typically 2 g / kg based on ideal body weight) should be used for the treatment of MIS-C (Figure 2). For patients without Kawasaki disease-like features, a lower dose is typically recommended (1 g / kg in 8 to 12 h). kg). The cardiac functions of patients, who are planned to use intravenous immunoglobulin, should be evaluated, and IVIG should be followed closely in patients with depressed cardiac functions, and diuretics should be administered if necessary. In addition, in some patients with cardiac dysfunction, IVIG may be given in divided doses (1 g / kg per day for 2 days). Low and medium doses of glucocorticoids (1–2mg / kg / day) should be given in two divided doses together with IVIG as adjunctive therapy in the treatment of patients with shock or life-threatening disease. It is recommended to administer high-dose intravenous glucocorticoid (10–30 mg / kg / day maximum 1 g) especially in patients who need high-dose inotropes or multiple inotropes and / or vasopressors if they do not respond to intravenous immunoglobulin and low-medium dose glucocorticoids. Low to medium dose steroids (1–2m / kg / day) may be considered in patients with milder forms of MIS-C with persistent fever and symptoms despite a single IVIG dose. Once the patient has recovered clinically, they can be switched to an equivalent oral prednisolone or prednisone dose at discharge and then reduced for three to four weeks. Anakinra (> 4 mg / kg / day IV or subcutaneous) treatment may be considered in patients who are unresponsive to MIS-C and MAS who are unresponsive to intravenous immunoglobulin and glucocorticoids and who are contraindicated to the use of long-term glucocorticoids. Serial laboratory tests and cardiac evaluation should guide immunomodulatory treatment response and reduction of therapy. Patients may need immunomodulatory medication for 2–3 weeks or even longer.

 In patients with MIS-C, low-dose aspirin (3–5 mg / kg / day; maximum 81 mg / day) can be used until the platelet count returns to normal, and normal coronary arteries are confirmed 4 weeks after diagnosis. Aspirin should be avoided in patients with active bleeding, significant bleeding risk and / or platelet count ≤ 80,000 / μL. However, patients with coronary artery aneurysm and a maximum z-score of 2.5–10 are treated with low-dose aspirin. In addition, patients with a Z-score of ≥ 10 should be treated with low-dose aspirin as well as therapeutic anticoagulation with enoxaparin (factor Xa level between 0.5–1) or warfarin. Longer use of enoxaparin dosage indications includes Z-score> 10 (indefinite treatment), with coronary artery aneurysm, documented thrombosis (≥3 months of treatment awaiting thrombus resolution), or moderate to severe ongoing left ventricular dysfunction.

 Antiplatelet therapy or anticoagulant treatment can be initiated in addition to IVIG in cases with incomplete or complete KD feature. If there are findings of coronary artery involvement, steroid therapy can be started. In patients with documented thrombosis or ejection fraction <35% should be given therapeutic anticoagulation with enoxaparin for at least 2 weeks after discharge from hospital.

Anakinra (recombinant human interleukin-1 receptor antagonist), canakinumab (human anti-interleukin-1β monoclonal antibody), and tocilizumab (anti-interleukin-6 receptor monoclonal antibody), in patients who are unable to receive glucocorticoids and who are resistant to glucocorticoids, are alternative options for treatment before mechanical ventilation therapy and macrophage activation syndrome. In the current case series, IL-1 and IL-6 inhibitors have been used in approximately 10%–20% of patients (19,34). Tocilizumab is not recommended in most pediatric patients because of its long half-life and lack of much benefit in studies in adults [40].

 Children who develop shock are treated according to shock protocols. Vasoconstrictor therapy such as adrenaline and noradrenaline can be given to the patient who is in the refractory shock clinic to fluid therapy. Adrenaline may be preferred especially in patients with left ventricular dysfunction. During periods, when the disease is intense, patients should be closely monitored for complications such as arrhythmia and embolism, and, if necessary, they should be followed up by repeated electrocardiogram, echocardiography, and cardiac enzyme monitoring.

 Patients with significant left ventricular dysfunction are treated with intravenous diuretics and agents such as milrinone, dopamine, and dobutamine. Mechanical hemodynamic support in the form of extracorporeal membrane oxygenation or a ventricular assist device may be required in cases of fulminant disease [32].

 Multiple systemic inflammatory syndrome may present with signs and symptoms mimicking septic shock and toxic shock syndrome (TSS). Therefore, patients with severe multisystem involvement, especially those with shock, should receive empirically broad-spectrum antibiotic therapy, while the culture results are awaited. An appropriate empirical regimen consists of ceftriaxone and vancomycin. Clindamycin can be added if there are toxin-mediated disease-like features. While SARS-CoV-2 antibody positivity is observed in most of the patients with multiple systemic inflammatory syndrome, RT-PCR positivity is seen in very few patients. Therefore, antiviral therapy may reduce the severity of the disease in selected RT-PCR positive cases.

## 9.Prognosis

Although multiple systemic inflammatory syndrome has many similarities to KD and toxic shock syndrome, the disease course in MIS-C can be more severe, and many children may require intensive care intervention. Most children survive, but there have been a few reported deaths [40]. In a systematic review of 16 case series, including a total of 655 MIS-C patients, 11 deaths (1.7%) occured [33].

 Patients with a diagnosis of multiple systemic inflammatory syndrome with abnormal BNP and / or troponin T levels are recommended to be followed up until these laboratory parameters reach normal values. Cardiac electrical conduction abnormalities are increasingly being reported in MIS-C. Therefore, in hospitalized MIS-C patients, an ECG should be taken at least every 48 h during the visit [39]. If there are conductive abnormalities as a result of the electrocardiogram, patients should be continuously monitored by cardiac monitoring, and follow-up with Holter ECG should be considered. Z scores indexed to body surface area should be measured using echocardiograms, ventricular / valve functions, pericardial infusion, and coronary artery dimensions during diagnosis and clinical follow-up. After discharge, children with MIS-C will need a close clinical follow-up with cardiology.

Approximateliy 20% of patients with cardiac involvement have continued mild suppression in cardiac functions during discharge from the hospital [41]. Long-term complications of myocardial inflammation should be carefully monitored for other forms of myocarditis. In the acute phase of the disease, patients who present with significant transient left ventricular dysfunction (left ventricular injection fraction <50%) or persistent left ventricular dysfunction are recommended to undergo cardiac magnetic resonance imaging after 2–6 months in order to show cardiac myocardial fibrosis and scar [32].
